# Net Charges of the Ribosomal Proteins of the *S10* and *spc* Clusters of Halophiles Are Inversely Related to the Degree of Halotolerance

**DOI:** 10.1128/spectrum.01782-21

**Published:** 2021-12-15

**Authors:** Madhan R. Tirumalai, Daniela Anane-Bediakoh, Sidharth Rajesh, George E. Fox

**Affiliations:** a Department of Biology and Biochemistry, University of Houston, Houston, Texas, USA; b Forensic Biology, The Houston Forensic Science Center, Houston, Texas, USA; c Clements High School (Class of 2023), Fort Bend Independent School District, Sugar Land, Texas, USA; Washington University in St. Louis

**Keywords:** net charges, ribosomal proteins, *S10-spc* cluster, halophiles, *S10-spc* operons/cluster

## Abstract

Net positive charge(s) on ribosomal proteins (r-proteins) have been reported to influence the assembly and folding of ribosomes. A high percentage of r-proteins from extremely halophilic archaea are known to be acidic or even negatively charged. Those proteins that remain positively charged are typically far less positively charged. Here, the analysis is extended to non-archaeal halophilic bacteria, eukaryotes, and halotolerant archaea. The net charges (pH 7.4) of the r-proteins that comprise the *S10-spc* operon/cluster from individual microbial and eukaryotic genomes were estimated and intercompared. It was observed that, as a general rule, the net charges of individual proteins remained mostly basic as the salt tolerance of the bacterial strains increased from 5 to 15%. The most striking exceptions were the extremely halophilic bacterial strains, Salinibacter ruber SD01, Acetohalobium arabaticum DSM 5501 and Selenihalanaerobacter shriftii ATCC BAA-73, which are reported to require a minimum of 18% to 21% salt for their growth. All three strains have higher numbers of acidic *S10-spc* cluster r-proteins than what is seen in the moderate halophiles or the halotolerant strains. Of the individual proteins, only uL2 never became acidic. uS14 and uL16 also seldom became acidic. The net negative charges on several of the *S10-spc* cluster r-proteins are a feature generally shared by all extremely halophilic archaea and bacteria. The *S10-spc* cluster r-proteins of halophilic fungi and algae (eukaryotes) were exceptions: these were positively charged despite the halophilicity of the organisms.

**IMPORTANCE** The net charges (at pH 7.4) of the ribosomal proteins (r-proteins) that comprise the *S10-spc* cluster show an inverse relationship with the halophilicity/halotolerance levels in both bacteria and archaea. In non-halophilic bacteria, the *S10-spc* cluster r-proteins are generally basic (positively charged), while the rest of the proteomes in these strains are generally acidic. On the other hand, the whole proteomes of the extremely halophilic strains are overall negatively charged, including the *S10-spc* cluster r-proteins. Given that the distribution of charged residues in the ribosome exit tunnel influences cotranslational folding, the contrasting charges observed in the *S10-spc* cluster r-proteins have potential implications for the rate of passage of these proteins through the ribosomal exit tunnel. Furthermore, the universal protein uL2, which lies in the oldest part of the ribosome, is always positively charged irrespective of the strain/organism it belongs to. This has implications for its role in the prebiotic context.

## INTRODUCTION

The ribosome is a universal molecular machine comprised of RNA and proteins (1) which catalyzes coded protein synthesis in all three domains of life (2–4). Thirty-four ribosomal proteins (r-proteins) are universally conserved (5–9). Of these, 21 are encoded by two large clusters which are analogous to the *S10* and *spc* operons in E. coli. These clusters contain four additional genes in *Archaea* and *Eukarya* (9). Given that RNA is negatively charged, the electrostatic properties of r-properties are expected to play a role in stabilizing r-protein-rRNA interactions in the ribosome structure.

In previous examinations of the electrostatic properties of r-proteins, it was observed that extremely halophilic archaeal r-proteins were observed in general to be negatively charged. This is in stark contrast with those from non-halophilic *Archaea* (10, 11). The proteomes of halophilic species are overrepresented by acidic residues (12, 13). It is thought that this may reflect a genetic adaptation. Earlier work by Kushner (1978) (14), Kushner and Kamekura (1988) (15), and Ventosa et al. (1998) (16), classified halophiles based on their salt requirement and tolerance limit(s). Moderate halophiles have been classified as those growing optimally between 0.5 M and 2.5 M salt (14, 15). Strains that can tolerate a broad range of low-high salt concentrations are classified as halotolerant. When growth is possible from low concentration and extends above 2.5 M, these strains are classified as extremely halotolerant. Halophiles that require at least 2 M salt for growth are considered extreme halophiles (15).

Despite the availability of cultured bacteria, archaea, fungi, and algae, and their characterized genomes/proteomes (16–32), little is known of the electrostatic properties of the r-proteins of halophilic and halotolerant bacteria, fungi, algae, and moderately halophilic archaea. These halophiles range from moderately halophilic, halotolerant, and extremely halotolerant to extremely obligately halophilic bacteria, fungi, and algae (16, 17, 19, 33–67). Additionally, there are several moderately halophilic archaea, including several methanogens (20, 39, 68, 69). On the other hand, extremely halophilic strains tend to be obligately halophilic, with minimum salt requirements of 18% to 21% (70). *A. arabaticum* (18, 71, 72), *S. shriftii* (73) and *S. ruber* (74), with salt requirements of 15% to 18% (71), 21% (73), and 20% to 25% (75), respectively (Table S1), are examples of extremely halophilic bacteria.

Here, the results of comparison of the net charges (pH 7.4) of *S10-spc* r-protein homologs from several halotolerant, extremely halophilic, and non-halophilic microbial (bacterial and archaeal) and eukaryotic genomes are reported. The results in part correlate with the extent of halotolerance.

## RESULTS

### Non-halophiles, including Archaea and Eukaryotes.

**FIG 1:**
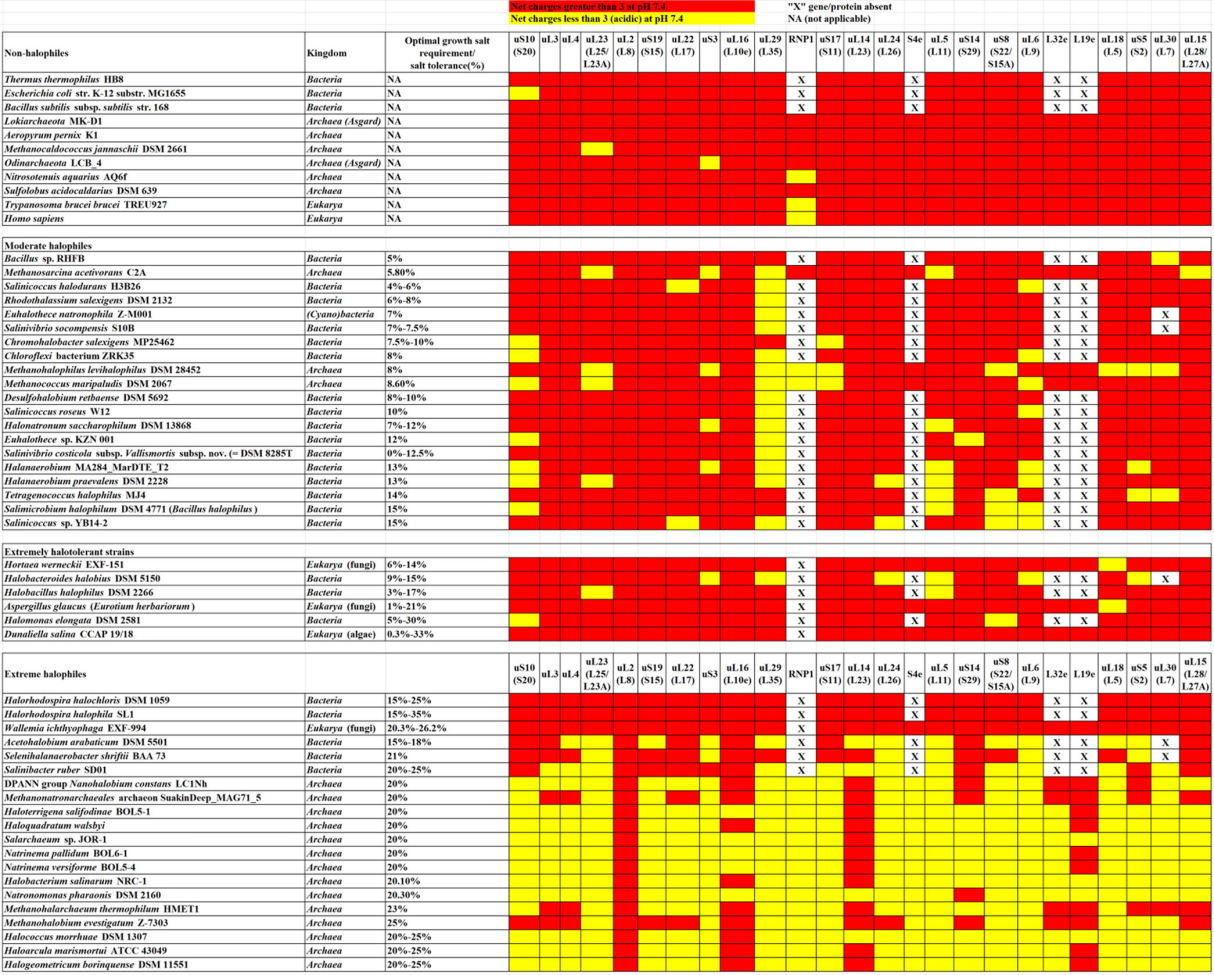
Net charges of the ribosomal proteins of the *S10-spc* cluster from representative strains of *Bacteria*, *Archaea*, and *Eukarya*. Proteins that have net charges (at pH 7.4) greater than 3 are shown in red, while those with net charges lesser than 3 are in yellow.

**FIG 2:**
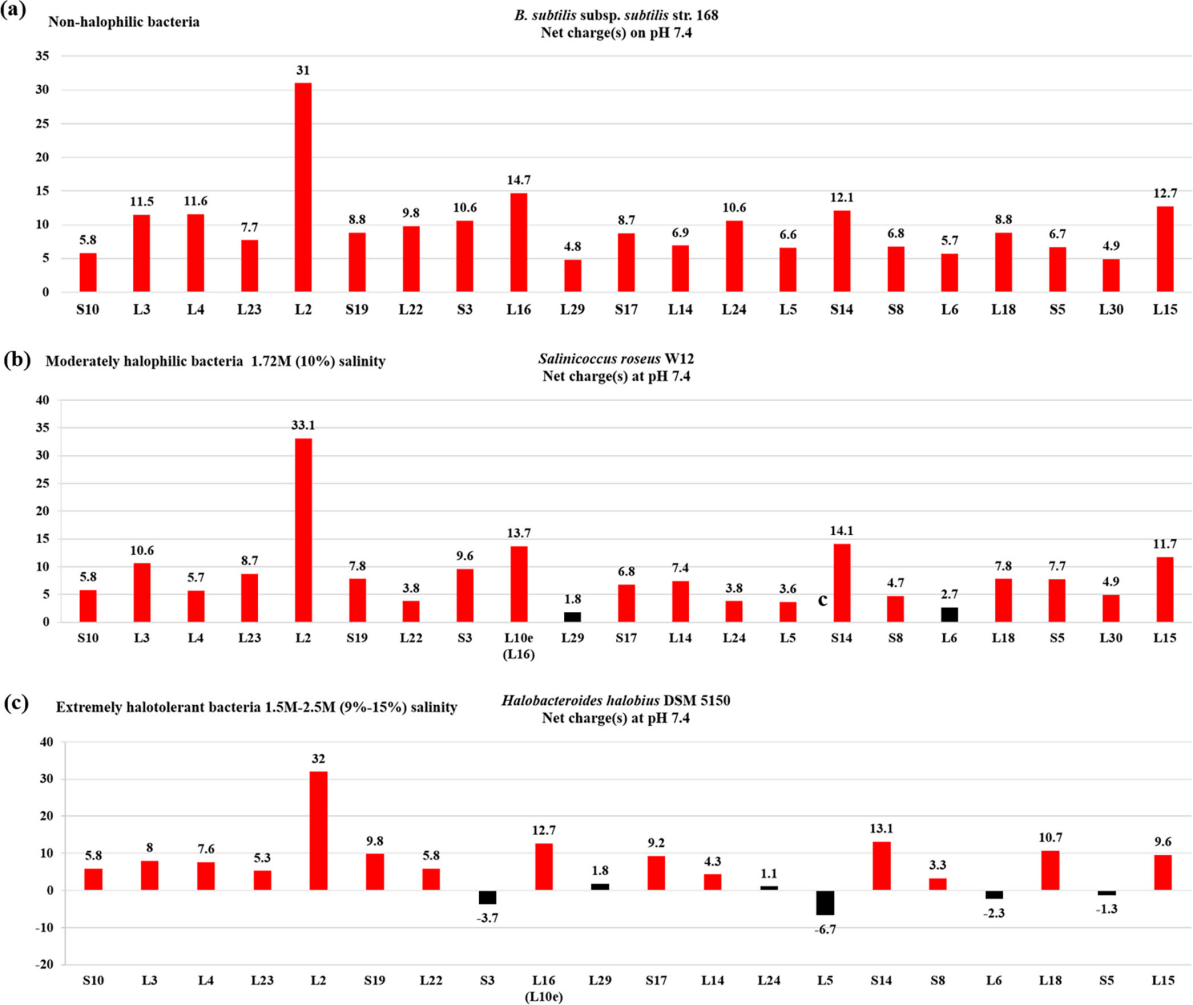
Net charges of the ribosomal proteins of the *S10-spc* cluster from representative strains of moderately halophilic Bacteria. (a) *B. subtilis* subsp. *subtilis* str. 168 (non-halophile). (b) *Salinicoccus roseus* W12 (10% salt). (c) *Halobacteroides halobius* DSM 5150 (9% to 15% salt). The charge value of each protein is shown for each bar; charges greater or lesser than 3 are shown in red and black, respectively.

**FIG 3:**
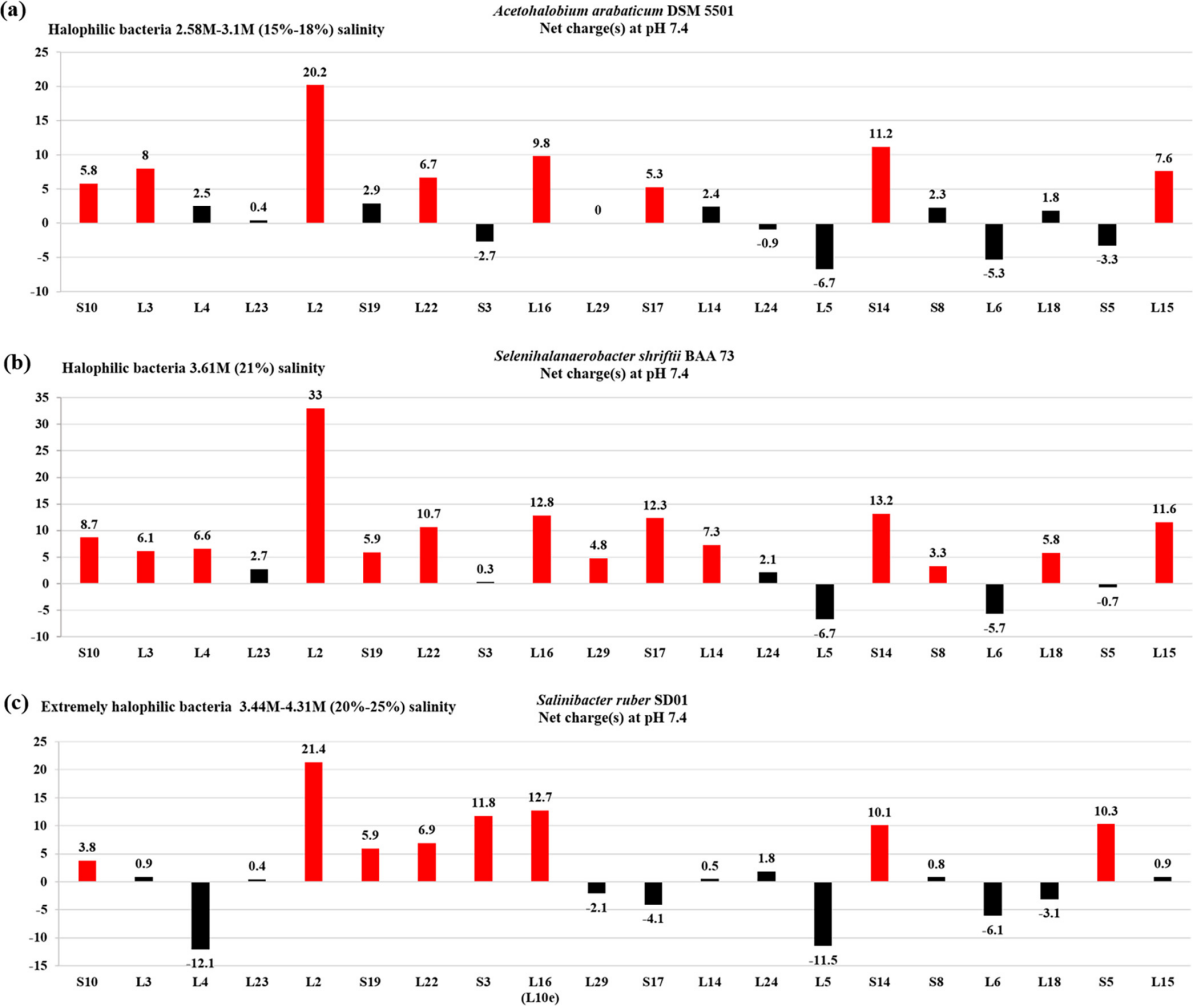
Net charges of the ribosomal proteins of the *S10-spc* cluster from representative strains of extremely halophilic bacteria. (a) Acetohalobium arabaticum DSM 5501 (15% to 18% salt). (b) Selenihalanaerobacter shriftii ATCC BAA-73 (21% salt). (c) Salinibacter ruber SD01 (20% to 25% salt). The charge value of each protein is shown for each bar; charges greater or lesser than 3 are shown in red and black, respectively.

**FIG 4:**
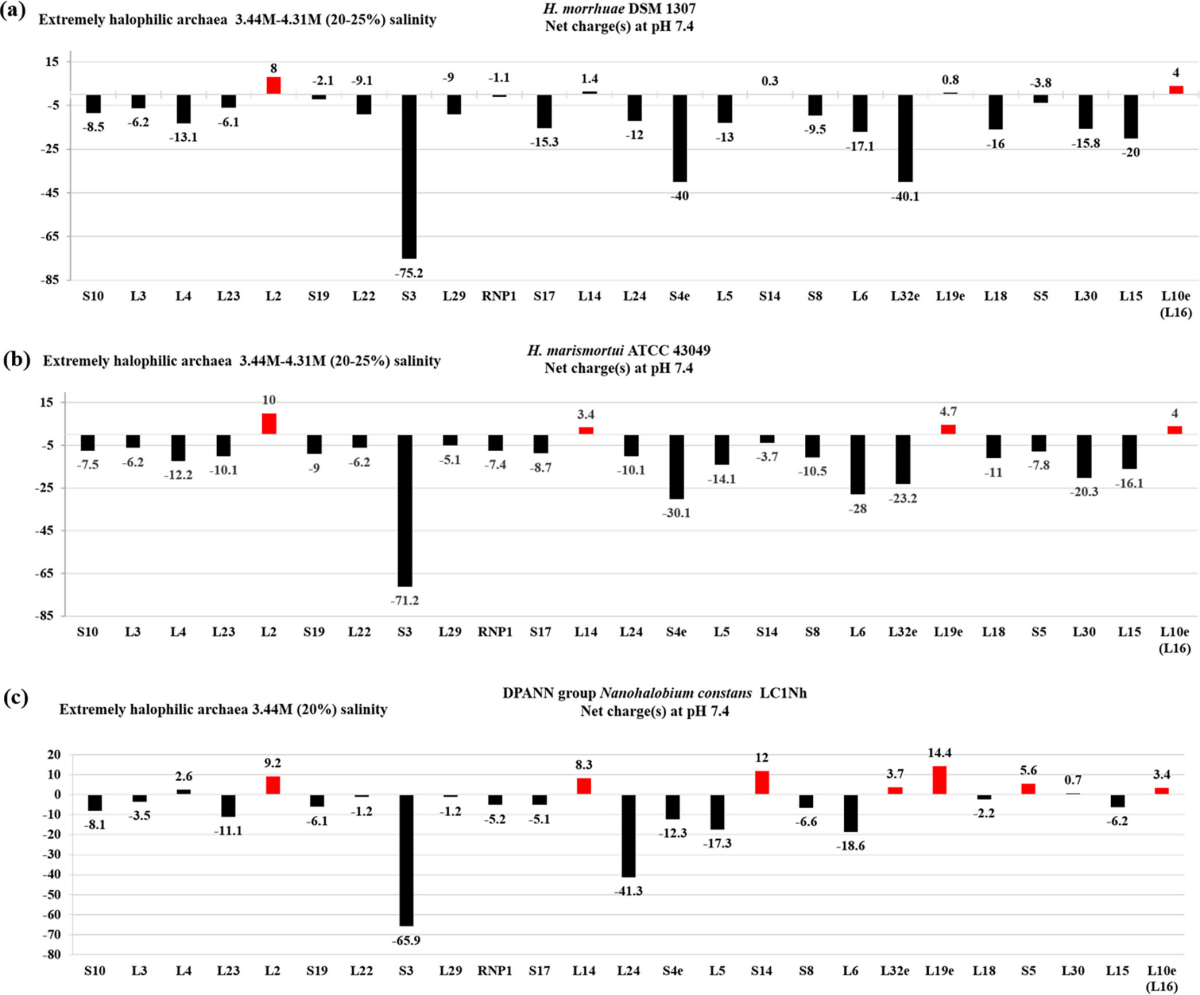
Net charges of the ribosomal proteins of the *S10-spc* cluster from representative strains of extremely halophilic Archaea. (a) *H. morrhuae* DSM 1307 (20% to 25% salt). (b) *H. marismortui* ATCC 43049 (20% to 25% salt). (c) DPANN group *Nanohalobium constans* LC1Nh (20% salt). The charge value of each protein is shown for each bar; charges greater or lesser than 3 are in red and black, respectively.

The charges on the r-proteins of the homolog equivalents of the *S10-spc* operon/cluster were examined and compared to each other in the data set of protein sequences from bacteria, archaea, and eukaryotes. In both bacteria and archaea, an increase in the salt tolerance limit is inversely related to the net charges on the r-proteins examined (Fig. 1–4). Based on the general principle that weak acidity ranges from pH 3 to 6 and strong acidity is pH <3 (76, 77), the cutoff pH for acidity of the charges was set at 3. As the level of tolerance/halophilicity goes above 15%, many of the r-proteins show charges that are less than 3.

### Extremely halophilic archaea.

In both the extremely halophilic bacteria and archaea examined, there is a significant increase in the number of *S10-spc* cluster r-proteins that are negatively charged or have charges of less than 3. Three extremely halophilic bacterial strains Salinibacter ruber SD01, Acetohalobium arabaticum DSM 5501, and Selenihalanaerobacter shriftii BAA-73 were part of this analysis (Fig. 3). Twelve of the 21 *S10-spc* cluster r-proteins in the strains *S. ruber* SD01 and *A. arabaticum* DSM 5501, and 6 of these proteins in the strain *S. shriftii* ATCC BAA-73, possess charges of less than 3 (acidic). The acidic properties of the *S10-spc* cluster r-proteins are shared by the halophilic archaea (Fig. 1).

Halotolerant/halophilic fungi (eukarya) and some halotolerant bacteria are exceptions to this. The bacterial strain *H. elongata* DSM 2581, which is tolerant to a broad range of salt concentrations (5% to 30%), is extremely halotolerant (78–80). The extremely halophilic bacterial strains Halorhodospira halochloris DSM 1059 and Halorhodospira halophila SL1 grow optimally at salt concentrations of 15% to 25% and 15% to 35%, respectively (81–84). In these three bacterial strains, all the *S10-spc* cluster r-proteins had charges that were greater than 3, except for uS10 and uS8 in *H. elongata* DSM 2581 (Fig. 1).

The extremely halotolerant fungal strains, namely Aspergillus *glaucus* (*Eurotium herbariorum*) and *Hortaea werneckii* EXF-151, the extremely halophilic fungal strain *W. ichthyophaga* EXF-994, and the extremely halotolerant algae *Dunaliella salina*, were all isolated from hypersaline environments (34, 55, 56, 65, 85–91). Except for uL18 in Aspergillus *glaucus* and *Hortaea werneckii* EXF-151,the net charges (pH 7.4) of the r-proteins of the *S10-spc* clusters in these organisms were all greater than 3 (Fig. 1 and Fig. S1 to S2).

The r-proteins of the *S10-spc* operon cluster in non-halophilic bacteria, non-halophilic archaea, and eukarya had positive charges (>3) (Fig. 1). The exceptions to this were the r-proteins uS10 (in the bacterium E. coli MG1655), uL23 (in archaeon *M. jannaschii* DSM 2661), uS3 (in the Asgard archaeon *Odinarchaeota* LCB_4), uL16 (L10e; in the eukaryote T. brucei *brucei* TREU927) and RNase P1 (RNP1 in the two eukaryotes examined), which had charges of less than 3 (Fig. 1).

Within the set of r-proteins of the *S10-spc* operon clusters examined, r-protein uL2 was uniquely positively charged (>3) irrespective of the species or the domain to which the species belonged. In bacteria, uL2 homologs had the highest charges compared to other cluster proteins. In bacteria, the charges of the uL2 homologs were consistently higher than those of the other proteins in the *S10-spc* cluster. The uL2 homolog with the lowest net charge of 5 (at pH 7.4) was found in the extremely halophilic archaeon *H. salinarum* NRC-1 (Table S2). This was also considered when setting the cutoff value for the net charge(s) of the proteins examined. Likewise, most uL14 homologs showed charges of >3, except for the homologs in the extreme halophiles *A. arabaticum* DSM 5501 (Bacteria), *S. ruber* SD01 (Bacteria), *N. pharaonis* DSM 2160 (Archaea), and *H. morrhuae* DSM 1307 (Archaea) (Fig. 1 and 2).

### Net charges on the non-r-proteins (*S10-spc* cluster).

The whole proteomes of the extremely halophilic strains were overall negatively charged, including the r-proteins. In contrast, an unusual pattern was observed in the proteomes of the non-halotolerant bacteria, such as E. coli and T. thermophilus, and of the moderately halophilic *Bacillus* sp. RHFB (92), *E. natronophila* Z-M001 (cyanobacteria) (41), and *S. roseus* (46). In these strains, while the *S10-spc* cluster proteins showed net positive charges (Fig. 1), the rest of the proteome showed negative charges (data not shown).

## DISCUSSION

### Salt tolerance and net charges of r-proteins.

In a previous analysis of the electrostatic properties of r-proteins from bacteria (E. coli, T. thermophilus, and D. radiodurans), halophilic archaea, and non-halophilic archaea, negative charges were uniquely found among the r-proteins of extremely halophilic archaea (10).

In this study, an inverse relationship was observed between the halotolerance limits of bacteria/archaea and the net charges of r-proteins of the highly conserved *S10-spc* cluster. In the moderately halophilic bacteria or archaea, which can tolerate up to 15% salt concentration, the charges on the *S10-spc* cluster r-proteins are less than those of their homologs in non-halotolerant bacterial strains. Halotolerance is most likely an outcome of properties such as the production of intracellular osmolytes, solutes, or salting-out strategies (70, 86, 93) to counterbalance the ionic imbalance in fluctuating ionic environments. However, as halotolerance extends above 15%, many of the *S10-spc* cluster r-proteins of extremely halophilic bacterial and archaeal strains show net negative charges, implying a genomic-level adaptation that is unique to these strains (Fig. 1 and 2). In fact, it has been suggested that the halophilic bacterial strain Salinibacter ruber SD01 is similar to the extremely halophilic archaeal strains Halobacterium salinarum and Haloarcula marismortui, both at the genomic and at the physiological level (94). This gene-level adaptation strategy is most likely an evolutionary outcome to minimize the energy expenditure required to survive in very high salt concentrations.

In contrast to the drastic changes in the net charges of the r-proteins of the *S10-spc* cluster in extremely halophilic bacteria and archaea, the homologs of this cluster in halophilic fungi and algae (eukaryotes) show positive charges (Fig. 1, Fig. S1 to S2). In an earlier study, it was observed that high levels of acidic residues were frequent in the protein families of three extremely halotolerant/halophilic fungal species: *W. ichthyophaga*, *H. werneckii*, and *E. rubrum* (95). Such strains have also been reported to use traits such as melanin-like pigment production, compatible solute production, ion efflux mechanisms, morphological changes, and regulation of plasma membrane fluidity to survive in hypersaline conditions (86, 96–99). These observations in halophilic eukaryotes suggest that adaptation to hypersaline conditions is likely a result of a combination of acidic residues in proteins, as well as changes in physiology and biochemistry. This is clearly not the case with the *S10-spc* cluster protein homologs of the halophilic eukaryotic strains examined in this study.

Of all the *S10-spc* cluster r-proteins, the net charge on uL2 always remains >3, irrespective of the organism/domain, despite a steady decrease in the charge(s) corresponding with an increase in halotolerance (Table S2). uL2 is a universal r-protein and is among the first set of large subunit proteins to be assembled into the ribosome (6, 100, 101). In the assembled ribosome structure, it is in very close proximity to what is considered the oldest part of the ribosome, namely, the Peptidyl Transferase Center (PTC) (4, 102, 103). It is also thought to be ancient in origin (104). The universality of the positive charge on all uL2 homologs has implications for the possible nature of its predecessor peptide in the prebiotic world. In the prebiotic scenario, a positively charged uL2 would have helped maximize stable adhesion/binding to the region surrounding the proto-PTC.

### Implications of net charges on translational rate(s) and ribosome stability.

It has been posited that adaptation to extreme environments, such as high salt, might involve structural alterations of proteins that do not affect their functions (105). The ribosome exit tunnel regulates translation and protein folding (106–108). Certain amino acid sequence segments are also known to stall ribosomes (109, 110). In a study on the proteomes of multiple organisms by Requião R.D. et al. (111), negatively charged proteins were found to be overrepresented. Thus, it was suggested that the charges on the nascent peptide are probably among the factors regulating translation efficiency and protein expression.

However, the vast differences in the net charges of many r-proteins of the *S10-spc* cluster between the non-halophiles, moderate halophiles, and extreme halophiles do not appear to significantly affect the ribosome tertiary structure. The core structure of the ribosome is shared by all three domains of life (5).

Furthermore, in studies on halophilic archaea, ribosome stability is known to be severely affected in low-salt concentration buffers (112, 113). However, despite the salt requirement for its stability, the overall basic structure of the ribosome in extreme halophilic archaea, such as *H. morrhuae* (113) or *H. marismortui* (114, 115), is similar to that found in bacteria. The amino acid residues are reported to undergo significant intermolecular segregation along the ribosomal proteins based on their charges. This has been shown to occur in such a manner as to have positively and negatively charged residues in buried and solvent export regions, respectively (10). With that said, the net low positive or negative charges of halophilic r-proteins found in extremely halophilic archaea/bacteria could be a major factor contributing to the high salt requirement for the stability of these ribosomes.

Finally, the distribution of charged residues in the ribosome exit tunnel (116) influences the landscape of cotranslational folding. For example, the positive charge density of r-proteins in E. coli is hypothesized to play a role in the cotranslational assembly of ribosomes by delaying the release of nascent r-proteins (117). Electrostatic interactions between positively charged residues (on the nascent peptide that is being synthesized) and the ribosomal tunnel are known to decrease translation rate (118). Therefore, the question is moot as to how negatively charged r-proteins of the *S10-spc* cluster in extreme halophiles affect the rate of the passage of the same through the negatively charged exit tunnel of the ribosome.

## CONCLUSIONS

The ribosome in general, and the exit tunnel in particular, are primarily negatively charged due to an RNA component which favors positively charged ribosomal proteins. Herein, the charges of the highly conserved ribosomal proteins found in the *S10-spc* gene cluster were used to characterize the extent of halotolerance in various microorganisms. It was expected and found that bacteria such as E. coli or B. subtilis that have little to no salt tolerance have the most positively charged proteins. Thus, the charges of the r-proteins of such bacteria are not markedly different from those of other non-halophilic archaea/eukarya. However, an increase in the salt tolerance limit results in a shift toward a more permanent change in the genome, resulting in encoding r-proteins with lower charges. This is evident in the net charge profiles of bacteria capable of growing optimally at salt concentrations above 15%. This trend is similar to that observed in extremely halophilic archaea. Individual proteins behave differently, with uL2 always remaining positive, which may reflect its role in holding the two subunits together. Contrasting charges on the r-proteins in bacteria/archaea may have implications for the passage of the growing protein through the exit tunnel and thus for the translation rate.

## MATERIALS AND METHODS

Protein and gene sequences from individual microbial (bacterial and archaeal) and eukaryotic organisms were downloaded from the public databases of the National Center for Biotechnology Information (NCBI) (119, 120). The net charges of all proteins (at pH 7.4) from each organism were estimated/calculated using the Isoelectric Point Calculator (121). The results were cross-verified with Prot Pi (https://www.protpi.ch/Calculator/ProteinTool) and Protein Calculator v. 3.4 (http://protcalc.sourceforge.net/). Additionally, the net charges (at pH 7.4) on the sequences of the r-proteins belonging to the equivalent of the *S10-spc* cluster from each genome were compared with those of the rest of the proteins in each genome.

### Bacterial, archaeal strains and eukaryotes used in comparisons.

A list of the organisms (and genomes) covered is given in Table S1 in the supplemental material.

### Data availability.

The data sets used and analyzed within the current study are available from the NCBI website as referenced in the paper.

## References

[1] Fox GE. 2016. Origins and early evolution of the ribosome, p 31–60. *In* Jagus GHR (ed), Evolution of the protein synthesis machinery and its regulation, 1 ed. Springer International Publishing AG. doi:10.1007/978-3-319-39468-8.

[2] Cech TR. 2000. Structural biology. The ribosome is a ribozyme. Science 289:878–879. doi:10.1126/science.289.5481.878. 10960319

[3] Beringer M, Rodnina MV. 2007. The ribosomal peptidyl transferase. Mol Cell 26:311–321. doi:10.1016/j.molcel.2007.03.015. 17499039

[4] Tirumalai MR, Rivas M, Tran Q, Fox GE. 2021. The peptidyl transferase center: a window to the past. Microbiol Mol Biol Rev 85:e00104-21. doi:10.1128/MMBR.00104-21. 34756086 PMC8579967

[5] Bowman JC, Petrov AS, Frenkel-Pinter M, Penev PI, Williams LD. 2020. Root of the tree: the significance, evolution, and origins of the ribosome. Chem Rev 120:4848–4878. doi:10.1021/acs.chemrev.9b00742. 32374986

[6] Lecompte O, Ripp R, Thierry JC, Moras D, Poch O. 2002. Comparative analysis of ribosomal proteins in complete genomes: an example of reductive evolution at the domain scale. Nucleic Acids Res 30:5382–5390. doi:10.1093/nar/gkf693. 12490706 PMC140077

[7] Yutin N, Puigbò P, Koonin EV, Wolf YI. 2012. Phylogenomics of prokaryotic ribosomal proteins. PLoS One 7:e36972. doi:10.1371/journal.pone.0036972. 22615861 PMC3353972

[8] Ban N, Beckmann R, Cate JH, Dinman JD, Dragon F, Ellis SR, Lafontaine DL, Lindahl L, Liljas A, Lipton JM, McAlear MA, Moore PB, Noller HF, Ortega J, Panse VG, Ramakrishnan V, Spahn CM, Steitz TA, Tchorzewski M, Tollervey D, Warren AJ, Williamson JR, Wilson D, Yonath A, Yusupov M. 2014. A new system for naming ribosomal proteins. Curr Opin Struct Biol 24:165–169. doi:10.1016/j.sbi.2014.01.002. 24524803 PMC4358319

[9] Wang J, Dasgupta I, Fox GE. 2009. Many nonuniversal archaeal ribosomal proteins are found in conserved gene clusters. Archaea 2:241–251. doi:10.1155/2009/971494. 19478915 PMC2686390

[10] Fedyukina DV, Jennaro TS, Cavagnero S. 2014. Charge segregation and low hydrophobicity are key features of ribosomal proteins from different organisms. J Biological Chemistry 289:6740–6750. doi:10.1074/jbc.M113.507707. PMC3945335 24398678

[11] Fukuchi S, Yoshimune K, Wakayama M, Moriguchi M, Nishikawa K. 2003. Unique amino acid composition of proteins in halophilic bacteria. J Mol Biol 327:347–357. doi:10.1016/s0022-2836(03)00150-5. 12628242

[12] Paul S, Bag SK, Das S, Harvill ET, Dutta C. 2008. Molecular signature of hypersaline adaptation: insights from genome and proteome composition of halophilic prokaryotes. Genome Biol 9:R70. doi:10.1186/gb-2008-9-4-r70. 18397532 PMC2643941

[13] Graziano G, Merlino A. 2014. Molecular bases of protein halotolerance. Biochim Biophys Acta 1844:850–858. doi:10.1016/j.bbapap.2014.02.018. 24590113

[14] Kushner DJ. 1978. Life in high salt and solute concentrations: halophilic bacteria, p 317–368. *In* Kushner DJ (ed), Microbial life in extreme environments. Academic Press, Ltd, London, United Kingdom.

[15] Kushner DJ, Kamekura M. 1988. Physiology of halophilic eubacteria, p 109–138. *In* Rodriguez-Valera F (ed), Halophilic bacteria, vol 1. CRC Press, Boca Raton.

[16] Ventosa A, Nieto JJ, Oren A. 1998. Biology of moderately halophilic aerobic bacteria. Microbiol Mol Biol Rev 62:504–544. doi:10.1128/MMBR.62.2.504-544.1998. 9618450 PMC98923

[17] Lowe SE, Jain MK, Zeikus JG. 1993. Biology, ecology, and biotechnological applications of anaerobic bacteria adapted to environmental stresses in temperature, pH, salinity, or substrates. Microbiol Rev 57:451–509. doi:10.1128/mr.57.2.451-509.1993. 8336675 PMC372919

[18] Zhilina TN, Zavarzin GA. 1990. Extremely halophilic, methylotrophic, anaerobic bacteria. FEMS Microbiology Lett 87:315–321. doi:10.1111/j.1574-6968.1990.tb04930.x.

[19] Ollivier B, Caumette P, Garcia JL, Mah RA. 1994. Anaerobic bacteria from hypersaline environments. Microbiol Rev 58:27–38. doi:10.1128/mr.58.1.27-38.1994. 8177169 PMC372951

[20] An BA, Shen Y, Voordouw J, Voordouw G. 2019. Halophilic methylotrophic methanogens may contribute to the high ammonium concentrations found in shale oil and shale gas reservoirs. Front Energy Res 7. doi:10.3389/fenrg.2019.00023.

[21] McGenity TJ, Sorokin DY. 2019. Methanogens and methanogenesis in hypersaline environments, p 283–309. *In* Stams AJM, Sousa DZ (ed), Biogenesis of hydrocarbons. Springer International Publishing, Cham, Switzerland. doi:10.1007/978-3-319-78108-2_12.

[22] Sorokin DY, Merkel AY, Abbas B, Makarova KS, Rijpstra WIC, Koenen M, Sinninghe Damste JS, Galinski EA, Koonin EV, van Loosdrecht MCM. 2018. *Methanonatronarchaeum thermophilum* gen. nov., sp. nov. and ‘Candidatus *Methanohalarchaeum thermophilum*’, extremely halo(natrono)philic methyl-reducing methanogens from hypersaline lakes comprising a new euryarchaeal class *Methanonatronarchaeia* classis nov. Int J Syst Evol Microbiol 68:2199–2208. doi:10.1099/ijsem.0.002810. 29781801 PMC6978985

[23] DasSarma P, Anton BP, DasSarma SL, von Ehrenheim HAL, Martinez FL, Guzmán D, Roberts RJ, DasSarma S. 2021. Genome sequence and methylation pattern of *Haloterrigena salifodinae* BOL5-1, an extremely halophilic archaeon from a Bolivian salt mine. Microbiol Resour Announc 10:e00275-21. doi:10.1128/MRA.00275-21. 33958400 PMC8103870

[24] Bolhuis H, Palm P, Wende A, Falb M, Rampp M, Rodriguez-Valera F, Pfeiffer F, Oesterhelt D. 2006. The genome of the square archaeon *Haloquadratum walsbyi*: life at the limits of water activity. BMC Genomics 7:169. doi:10.1186/1471-2164-7-169. 16820047 PMC1544339

[25] Anton Brian P, DasSarma P, Martinez Fabiana L, DasSarma Satyajit L, Al Madadha M, Roberts Richard J, DasSarma S, Stedman Kenneth M. 2020. Genome sequence of *Salarchaeum* sp. strain JOR-1, an extremely halophilic archaeon from the Dead Sea. Microbiol Resour Announc 9:e01505-19. doi:10.1128/MRA.01505-19. 32001568 PMC6992872

[26] DasSarma P, Anton BP, DasSarma SL, Martinez FL, Guzman D, Roberts RJ, DasSarma S, Stewart FJ. 2019. Genome sequences and methylation patterns of *Natrinema versiforme* BOL5-4 and *Natrinema pallidum* BOL6-1, two extremely halophilic Archaea from a Bolivian salt mine. Microbiol Resour Announc 8:e00810-19. doi:10.1128/MRA.00810-19. 31416876 PMC6696651

[27] Ng WV, Kennedy SP, Mahairas GG, Berquist B, Pan M, Shukla HD, Lasky SR, Baliga NS, Thorsson V, Sbrogna J, Swartzell S, Weir D, Hall J, Dahl TA, Welti R, Goo YA, Leithauser B, Keller K, Cruz R, Danson MJ, Hough DW, Maddocks DG, Jablonski PE, Krebs MP, Angevine CM, Dale H, Isenbarger TA, Peck RF, Pohlschroder M, Spudich JL, Jung KW, Alam M, Freitas T, Hou S, Daniels CJ, Dennis PP, Omer AD, Ebhardt H, Lowe TM, Liang P, Riley M, Hood L, DasSarma S. 2000. Genome sequence of *Halobacterium* species NRC-1. Proc Natl Acad Sci USA 97:12176–12181. doi:10.1073/pnas.190337797. 11016950 PMC17314

[28] Falb M, Pfeiffer F, Palm P, Rodewald K, Hickmann V, Tittor J, Oesterhelt D. 2005. Living with two extremes: conclusions from the genome sequence of *Natronomonas pharaonis*. Genome Res 15:1336–1343. doi:10.1101/gr.3952905. 16169924 PMC1240075

[29] Sorokin DY, Makarova KS, Abbas B, Ferrer M, Golyshin PN, Galinski EA, Ciordia S, Mena MC, Merkel AY, Wolf YI, van Loosdrecht MCM, Koonin EV. 2017. Discovery of extremely halophilic, methyl-reducing euryarchaea provides insights into the evolutionary origin of methanogenesis. Nat Microbiol 2:17081–17081. doi:10.1038/nmicrobiol.2017.81. 28555626 PMC5494993

[30] Ishida Y, Inouye K, Ming O, Inouye M. 2019. A CUGGU/UUGGU-specific MazF homologue from *Methanohalobium evestigatum*. Biochem Biophys Res Commun 518:533–540. doi:10.1016/j.bbrc.2019.08.076. 31445700

[31] Oren A, Ginzburg M, Ginzburg BZ, Hochstein LI, Volcani BE. 1990. *Haloarcula marismortui* (Volcani) sp. nov., nom. rev., an extremely halophilic bacterium from the Dead Sea. Int J Syst Bacteriol 40:209–210. doi:10.1099/00207713-40-2-209. 11536469

[32] Montalvo-Rodríguez R, Vreeland RH, Oren A, Kessel M, Betancourt C, López-Garriga J. 1998. *Halogeometricum borinquense* gen. nov., sp. nov., a novel halophilic archaeon from Puerto Rico. Int J Syst Bacteriol 48 Pt 4:1305–1312. doi:10.1099/00207713-48-4-1305. 9828431

[33] Ghozlan H, Deif H, Kandil RA, Sabry S. 2006. Biodiversity of moderately halophilic bacteria in hypersaline habitats in Egypt. J Gen Appl Microbiol 52:63–72. doi:10.2323/jgam.52.63. 16778349

[34] Zajc J, Zalar P, Plemenitaš A, Gunde-Cimerman N. 2012. The mycobiota of the salterns. Prog Mol Subcell Biol 53:133–158. doi:10.1007/978-3-642-23342-5_7. 22222830

[35] Michoud G, Ngugi DK, Barozzi A, Merlino G, Calleja ML, Delgado-Huertas A, Morán XAG, Daffonchio D. 2021. Fine-scale metabolic discontinuity in a stratified prokaryote microbiome of a Red Sea deep halocline. ISME J 15:2351–2365. doi:10.1038/s41396-021-00931-z. 33649556 PMC8319295

[36] Copeland A, O'Connor K, Lucas S, Lapidus A, Berry KW, Detter JC, Del Rio TG, Hammon N, Dalin E, Tice H, Pitluck S, Bruce D, Goodwin L, Han C, Tapia R, Saunders E, Schmutz J, Brettin T, Larimer F, Land M, Hauser L, Vargas C, Nieto JJ, Kyrpides NC, Ivanova N, Göker M, Klenk HP, Csonka LN, Woyke T. 2011. Complete genome sequence of the halophilic and highly halotolerant *Chromohalobacter salexigens* type strain (1H11 (T)). Stand Genomic Sci 5:379–388. doi:10.4056/sigs.2285059. 22675587 PMC3368415

[37] Arahal DR, García MT, Vargas C, Cánovas D, Nieto JJ, Ventosa A. 2001. *Chromohalobacter salexigens* sp. nov., a moderately halophilic species that includes *Halomonas elongata* DSM 3043 and ATCC 33174. Int J Syst Evol Microbiol 51:1457–1462. doi:10.1099/00207713-51-4-1457. 11491346

[38] Meier DV, Greve AJ, Chennu A, van Erk MR, Muthukrishnan T, Abed RMM, Woebken D, de Beer D. 2021. Limitation of microbial processes at saturation-level salinities in a microbial mat covering a coastal salt flat. Appl Environ Microbiol 87:e0069821. doi:10.1128/AEM.00698-21. 34160273 PMC8357274

[39] Maestrojuan GM, Boone JE, Mah RA, Menaia JAGF, Sachs MS, Boone DR. 1992. Taxonomy and halotolerance of mesophilic *Methanosarcina* strains, assignment of strains to species, and synonymy of *Methanosarcina mazei* and *Methanosarcina frisia*. Int J Syst Evol Microbiol 42:561–567. doi:10.1099/00207713-42-4-561.

[40] Venkata Ramana V, Kalyana Chakravarthy S, Ramaprasad EV, Thiel V, Imhoff JF, Sasikala C, Ramana CV. 2013. Emended description of the genus *Rhodothalassium* Imhoff et al., 1998 and proposal of *Rhodothalassiaceae* fam. nov. and *Rhodothalassiales* ord. Syst Appl Microbiol 36:28–32. doi:10.1016/j.syapm.2012.09.003. 23265196

[41] Mikhodiuk OS, Gerasimenko LM, Akimov VN, Ivanovskiĭ RN, Zavarzin GA. 2008. Ecophysiology and polymorphism of the unicellular extremely natronophilic cyanobacterium *Euhalothece* sp. Z-M001 from Lake Magadi. Mikrobiologiia 77:805–813. 19137720

[42] Galisteo C, Sánchez-Porro C, de la Haba RR, López-Hermoso C, Fernández AB, Farias ME, Ventosa A. 2019. Characterization of *Salinivibrio socompensis* sp. nov., a new halophilic bacterium isolated from the high-altitude hypersaline Lake Socompa, Argentina. Microorganisms 7:241. doi:10.3390/microorganisms7080241. 31387286 PMC6723482

[43] Katayama T, Yoshioka H, Mochimaru H, Meng XY, Muramoto Y, Usami J, Ikeda H, Kamagata Y, Sakata S. 2014. *Methanohalophilus levihalophilus* sp. nov., a slightly halophilic, methylotrophic methanogen isolated from natural gas-bearing deep aquifers, and emended description of the genus *Methanohalophilus*. Int J Syst Evol Microbiol 64:2089–2093. doi:10.1099/ijs.0.063677-0. 24670897

[44] Apolinario EA, Sowers KR. 1996. Plate colonization of *Methanococcus maripaludis* and *Methanosarcina thermophila* in a modified canning jar. FEMS Microbiology Lett 145:131–137. doi:10.1111/j.1574-6968.1996.tb08567.x.

[45] Jones WJ, Paynter MJB, Gupta R. 1983. Characterization of *Methanococcus maripaludis* sp. nov., a new methanogen isolated from salt marsh sediment. Arch Microbiol 135:91–97. doi:10.1007/BF00408015.

[46] Wang H, Wang L, Yang H, Cai Y, Sun L, Xue Y, Yu B, Ma Y. 2015. Comparative proteomic insights into the lactate responses of halophilic *Salinicoccus roseus* W12. Sci Rep 5:13776. doi:10.1038/srep13776. 26358621 PMC4566078

[47] Ventosa A, García MT, Kamekura M, Onishi H, Ruiz-Berraquero F. 1989. *Bacillus halophilus* sp. nov., a moderately halophilic *Bacillus* species. Syst Appl Microbiol 12:162–166. doi:10.1016/S0723-2020(89)80009-8.

[48] Yoon J-H, Kang S-J, Oh T-K. 2007. Reclassification of *Marinococcus albus* Hao et al. 1985 as *Salimicrobium album* gen. nov., comb. nov. and *Bacillus halophilus* Ventosa et al. 1990 as *Salimicrobium halophilum* comb. nov., and description of *Salimicrobium luteum* sp. nov. Int J Syst Evol Microbiol 57:2406–2411. doi:10.1099/ijs.0.65003-0. 17911318

[49] Jakobsen TF, Kjeldsen KU, Ingvorsen K. 2006. *Desulfohalobium utahense* sp. nov., a moderately halophilic, sulfate-reducing bacterium isolated from Great Salt Lake. Int J Syst Evol Microbiol 56:2063–2069. doi:10.1099/ijs.0.64323-0. 16957100

[50] Zhilina TN, Garnova ES, Turova TP, Kostrikina NA, Zavarzin GA. 2001. *Halonatronum saccharophilum* gen. nov. sp. nov.: a new haloalkalophilic bacteria from the order *Haloanaerobiales* from Lake Magadi. Mikrobiologiia 70:77–85. 11338841

[51] Huang CY, Garcia JL, Patel BK, Cayol JL, Baresi L, Mah RA. 2000. *Salinivibrio costicola* subsp. *vallismortis* subsp. nov., a halotolerant facultative anaerobe from Death Valley, and emended description of *Salinivibrio costicola*. Int J Syst Evol Microbiol 50:615–622. doi:10.1099/00207713-50-2-615. 10758867

[52] Zeikus JG, Hegge PW, Thompson TE, Phelps TJ, Langworthy TA. 1983. Isolation and description of *Haloanaerobium praevalens* gen. nov. and sp. nov., an obligately anaerobic halophile common to Great Salt Lake sediments. Curr Microbiol 9:225–233. doi:10.1007/BF01567586.

[53] Heo S, Lee J, Lee JH, Jeong DW. 2019. Genomic insight into the salt tolerance of *Enterococcus faecium*, *Enterococcus faecalis* and *Tetragenococcus halophilus*. J Microbiol Biotechnol 29:1591–1602. doi:10.4014/jmb.1908.08015. 31546297

[54] Jeong D-W, Heo S, Lee J-H. 2017. Safety assessment of *Tetragenococcus halophilus* isolates from doenjang, a Korean high-salt-fermented soybean paste. Food Microbiol 62:92–98. doi:10.1016/j.fm.2016.10.012. 27889172

[55] Zalar P, Zupancic J, Gostincar C, Zajc J, de Hoog GS, De Leo F, Azua-Bustos A, Gunde-Cimerman N. 2019. The extremely halotolerant black yeast *Hortaea werneckii*: a model for intraspecific hybridization in clonal fungi. IMA Fungus 10:10. doi:10.1186/s43008-019-0007-5. 32647617 PMC7325687

[56] Oren A. 2014. The ecology of *Dunaliella* in high-salt environments. J Biol Res (Thessalon) 21:23. doi:10.1186/s40709-014-0023-y. 25984505 PMC4389652

[57] Chen H, Jiang J-G, Wu G-H. 2009. Effects of salinity changes on the growth of *Dunaliella salina* and its isozyme activities of glycerol-3-phosphate dehydrogenase. J Agric Food Chem 57:6178–6182. doi:10.1021/jf900447r. 19548674

[58] Mogany T, Swalaha FM, Allam M, Mtshali PS, Ismail A, Kumari S, Bux F. 2018. Phenotypic and genotypic characterisation of an unique indigenous hypersaline unicellular cyanobacterium, *Euhalothece* sp.nov. Microbiol Res 211:47–56. doi:10.1016/j.micres.2018.04.001. 29705205

[59] Ouyang B, Akob DM, Dunlap DS, Renock D. 2017. Microbially mediated barite dissolution in anoxic brines. Appl Geochem 76:51–59. doi:10.1016/j.apgeochem.2016.11.008.

[60] Mevada V, Patel S, Pandya J, Joshi H, Patel R. 2017. Whole genome sequencing and annotation of halophilic *Salinicoccus* sp. BAB 3246 isolated from the coastal region of Gujarat. Genom Data 13:30–34. doi:10.1016/j.gdata.2017.06.006. 28702355 PMC5485554

[61] Oren A, Weisburg WG, Kessel M, Woese CR. 1984. *Halobacteroides halobius* gen. nov., sp. nov., a moderately halophic anaerobic bacterium from the bottom sediments of the Dead Sea. Syst Appl Microbiol 5:58–70. doi:10.1016/S0723-2020(84)80051-X.

[62] Jiang K, Xue Y, Ma Y. 2015. Complete genome sequence of *Salinicoccus halodurans* H3B36, isolated from the Qaidam Basin in China. Stand Genomic Sci 10:116–116. doi:10.1186/s40793-015-0108-8. 26634017 PMC4667468

[63] Saum SH, Pfeiffer F, Palm P, Rampp M, Schuster SC, Müller V, Oesterhelt D. 2013. Chloride and organic osmolytes: a hybrid strategy to cope with elevated salinities by the moderately halophilic, chloride-dependent bacterium *Halobacillus halophilus*. Environ Microbiol 15:1619–1633. doi:10.1111/j.1462-2920.2012.02770.x. 22583374

[64] Preisig H. 1992. Morphology and taxonomy, p 1–15. *In* Ben-Amotz A, Avron M (ed), Dunaliella: physiology, biochemistry, and biotechnology. CRC Press, Boca Raton, FL.

[65] Polle JEW, Barry K, Cushman J, Schmutz J, Tran D, Hathwaik LT, Yim WC, Jenkins J, McKie-Krisberg Z, Prochnik S, Lindquist E, Dockter RB, Adam C, Molina H, Bunkenborg J, Jin E, Buchheim M, Magnuson J. 2017. Draft nuclear genome sequence of the halophilic and beta-carotene-accumulating green alga *Dunaliella salina* strain CCAP19/18. Genome Announc 5:e01105-17. doi:10.1128/genomeA.01105-17. 29074648 PMC5658486

[66] Rainey FA, Zhilina TN, Boulygina ES, Stackebrandt E, Tourova TP, Zavarzin GA. 1995. The taxonomic status of the fermentative halophilic anaerobic bacteria: description of *Haloanaerobiales* ord. nov., *Halobacteroidaceae* fam. nov., *Orenia* gen. nov. and further taxonomic rearrangements at the genus and species level. Anaerobe 1:185–199. doi:10.1006/anae.1995.1018. 16887527

[67] Narasingarao P, Podell S, Ugalde JA, Brochier-Armanet C, Emerson JB, Brocks JJ, Heidelberg KB, Banfield JF, Allen EE. 2012. *De novo* metagenomic assembly reveals abundant novel major lineage of *Archaea* in hypersaline microbial communities. ISME J 6:81–93. doi:10.1038/ismej.2011.78. 21716304 PMC3246234

[68] Sowers KR, Boone JE, Gunsalus RP. 1993. Disaggregation of *Methanosarcina* spp. and growth as single cells at elevated osmolarity. Appl Environ Microbiol 59:3832–3839. doi:10.1128/aem.59.11.3832-3839.1993. 16349092 PMC182538

[69] Bowers KJ, Wiegel J. 2011. Temperature and pH optima of extremely halophilic archaea: a mini-review. Extremophiles 15:119–128. doi:10.1007/s00792-010-0347-y. 21340748

[70] Oren A. 2002. Molecular ecology of extremely halophilic *Archaea* and *Bacteria*. FEMS Microbiol Ecol 39:1–7. doi:10.1111/j.1574-6941.2002.tb00900.x. 19709178

[71] Sikorski J, Lapidus A, Chertkov O, Lucas S, Copeland A, Glavina Del Rio T, Nolan M, Tice H, Cheng JF, Han C, Brambilla E, Pitluck S, Liolios K, Ivanova N, Mavromatis K, Mikhailova N, Pati A, Bruce D, Detter C, Tapia R, Goodwin L, Chen A, Palaniappan K, Land M, Hauser L, Chang YJ, Jeffries CD, Rohde M, Göker M, Spring S, Woyke T, Bristow J, Eisen JA, Markowitz V, Hugenholtz P, Kyrpides NC, Klenk HP. 2010. Complete genome sequence of *Acetohalobium arabaticum* type strain (Z-7288). Stand Genomic Sci 3:57–65. doi:10.4056/sigs.1062906. 21304692 PMC3035264

[72] Zhilina TN, Zavarzin GA. 1990. A new extremely halophilic homoacetogen bacteria *Acetohalobium arabaticum*, gen. nov. sp. nov. Dokl Akad Nauk SSSR 311:745–747.

[73] Blum JS, Stolz JF, Oren A, Oremland RS. 2001. *Selenihalanaerobacter shriftii* gen. nov., sp. nov., a halophilic anaerobe from Dead Sea sediments that respires selenate. Arch Microbiol 175:208–219. doi:10.1007/s002030100257. 11357513

[74] Antón J, Oren A, Benlloch S, Rodríguez-Valera F, Amann R, Rosselló-Mora R. 2002. *Salinibacter ruber* gen. nov., sp. nov., a novel, extremely halophilic member of the *Bacteria* from saltern crystallizer ponds. Int J Syst Evol Microbiol 52:485–491. doi:10.1099/00207713-52-2-485. 11931160

[75] Bardavid RE, Ionescu D, Oren A, Rainey FA, Hollen BJ, Bagaley DR, Small AM, McKay C. 2007. Selective enrichment, isolation and molecular detection of *Salinibacter* and related extremely halophilic bacteria from hypersaline environments. Hydrobiologia 576:3–13. doi:10.1007/s10750-006-0288-8.

[76] Housecroft CE, Sharpe AG. 2004. Inorganic chemistry, 2nd ed Prentice Hall, Hoboken, NJ.

[77] Talley K, Alexov E. 2010. On the pH-optimum of activity and stability of proteins. Proteins 78:2699–2706. doi:10.1002/prot.22786. 20589630 PMC2911520

[78] Vreeland RH, Litchfield CD, Martin EL, Elliot E. 1980. *Halomonas elongata*, a new genus and species of extremely salt-tolerant bacteria. Int J Syst Evol Microbiol 30:485–495.

[79] Kindzierski V, Raschke S, Knabe N, Siedler F, Scheffer B, Pflüger-Grau K, Pfeiffer F, Oesterhelt D, Marin-Sanguino A, Kunte H-J. 2017. Osmoregulation in the halophilic bacterium *Halomonas elongata*: a case study for integrative systems biology. PLoS One 12:e0168818. doi:10.1371/journal.pone.0168818. 28081159 PMC5231179

[80] Vreeland RH. 1992. The family *Halomonadaceae*, p 3181–3188. *In* Balows A, Trüper HG, Dworkin M, Harder W, Schleifer KH (ed), The prokaryotes doi:10.1007/978-1-4757-2191-1_5. Springer, New York.

[81] Imhoff JF, Trüper HG. 1977. *Ectothiorhodospira halochloris* sp. nov., a new extremely halophilic phototrophic bacterium containing bacteriochlorophyll b. Arch Microbiol 114:115–121. doi:10.1007/BF00410772.

[82] Raymond JC, Sistrom WR. 1969. *Ectothiorhodospira halophila*: a new species of the genus *Ectothiorhodospira*. Arch Mikrobiol 69:121–126. doi:10.1007/BF00409756. 4192367

[83] Challacombe JF, Majid S, Deole R, Brettin TS, Bruce D, Delano SF, Detter JC, Gleasner CD, Han CS, Misra M, Reitenga KG, Mikhailova N, Woyke T, Pitluck S, Nolan M, Land ML, Saunders E, Tapia R, Lapidus A, Ivanova N, Hoff WD. 2013. Complete genome sequence of *Halorhodospira halophila* SL1. Stand Genomic Sci 8:206–214. doi:10.4056/sigs.3677284. 23991253 PMC3746430

[84] Deole R, Challacombe J, Raiford DW, Hoff WD. 2013. An extremely halophilic proteobacterium combines a highly acidic proteome with a low cytoplasmic potassium content. J Biol Chem 288:581–588. doi:10.1074/jbc.M112.420505. 23144460 PMC3537055

[85] Gunde-Cimerman N, Ramos J, Plemenitaš A. 2009. Halotolerant and halophilic fungi. Mycol Res 113:1231–1241. doi:10.1016/j.mycres.2009.09.002. 19747974

[86] Gunde-Cimerman N, Plemenitaš A, Oren A. 2018. Strategies of adaptation of microorganisms of the three domains of life to high salt concentrations. FEMS Microbiol Rev 42:353–375. doi:10.1093/femsre/fuy009. 29529204

[87] Rabinowitch S, Grover NB, Ginzburg BZ. 1975. Cation effects on volume and water permeability in the halophilic algae *Dunaliella parva*. J Membr Biol 22:211–230. doi:10.1007/BF01868172. 1159777

[88] Qiu W, Li J, Wei Y, Fan F, Jiang J, Liu M, Han X, Tian C, Zhang S, Zhuo R. 2020. Genome sequencing of *Aspergillus glaucus* ‘CCHA’ provides insights into salt-stress adaptation. PeerJ 8:e8609. doi:10.7717/peerj.8609. 32140304 PMC7045888

[89] Butinar L, Sonjak S, Zalar P, Plemenitaš A, Gunde-Cimerman N. 2005. Melanized halophilic fungi are eukaryotic members of microbial communities in hypersaline waters of solar salterns. Botanica Marina 48:73–79. doi:10.1515/BOT.2005.007.

[90] Plemenitaš A, Lenassi M, Konte T, Kejžar A, Zajc J, Gostinčar C, Gunde-Cimerman N. 2014. Adaptation to high salt concentrations in halotolerant/halophilic fungi: a molecular perspective. Front Microbiol 5:199. doi:10.3389/fmicb.2014.00199. 24860557 PMC4017127

[91] Zajc J, Kogej T, Galinski EA, Ramos J, Gunde-Cimerman N. 2014. Osmoadaptation strategy of the most halophilic fungus, *Wallemia ichthyophaga*, growing optimally at salinities above 15% NaCl. Appl Environ Microbiol 80:247–256. doi:10.1128/AEM.02702-13. 24162565 PMC3911034

[92] Petrillo C, Castaldi S, Lanzilli M, Selci M, Cordone A, Giovannelli D, Isticato R. 2021. Genomic and physiological characterization of Bacilli isolated from salt-pans with plant growth promoting features. Front Microbiol 12:715678. doi:10.3389/fmicb.2021.715678. 34589073 PMC8475271

[93] Oren A. 1999. Bioenergetic aspects of halophilism. Microbiol Mol Biol Rev 63:334–348. doi:10.1128/MMBR.63.2.334-348.1999. 10357854 PMC98969

[94] Oren A, Mana L. 2002. Amino acid composition of bulk protein and salt relationships of selected enzymes of *Salinibacter ruber*, an extremely halophilic bacterium. Extremophiles 6:217–223. doi:10.1007/s007920100241. 12072957

[95] Kis-Papo T, Weig AR, Riley R, Peršoh D, Salamov A, Sun H, Lipzen A, Wasser SP, Rambold G, Grigoriev IV, Nevo E. 2014. Genomic adaptations of the halophilic Dead Sea filamentous fungus *Eurotium rubrum*. Nat Commun 5:3745. doi:10.1038/ncomms4745. 24811710

[96] Gunde-Cimerman N, Zalar P. 2014. Extremely halotolerant and halophilic fungi inhabit brine in solar salterns around the globe. Food Technol and Biotechnol 52:170–179.

[97] Petrovic U, Gunde-Cimerman N, Plemenitas A. 2002. Cellular responses to environmental salinity in the halophilic black yeast *Hortaea werneckii*. Mol Microbiol 45:665–672. doi:10.1046/j.1365-2958.2002.03021.x. 12139614

[98] Kogej T, Ramos J, Plemenitaš A, Gunde-Cimerman N. 2005. The halophilic fungus *Hortaea werneckii* and the halotolerant fungus *Aureobasidium pullulans* maintain low intracellular cation concentrations in hypersaline environments. Appl Environ Microbiol 71:6600–6605. doi:10.1128/AEM.71.11.6600-6605.2005. 16269687 PMC1287720

[99] Ding X, Liu K, Lu Y, Gong G. 2019. Morphological, transcriptional, and metabolic analyses of osmotic-adapted mechanisms of the halophilic *Aspergillus montevidensis* ZYD4 under hypersaline conditions. Appl Microbiol and Biotechnol 103:3829–3846. doi:10.1007/s00253-019-09705-2. 30859256

[100] Fox GE. 2010. Origin and evolution of the ribosome. Cold Spring Harb Perspect Biol 2:a003483. doi:10.1101/cshperspect.a003483. 20534711 PMC2926754

[101] Rohl R, Nierhaus KH. 1982. Assembly map of the large subunit (50S) of *Escherichia coli* ribosomes. Proc Natl Acad Sci USA 79:729–733. doi:10.1073/pnas.79.3.729. 7038683 PMC345825

[102] Nissen P, Hansen J, Ban N, Moore PB, Steitz TA. 2000. The structural basis of ribosome activity in peptide bond synthesis. Science 289:920–930. doi:10.1126/science.289.5481.920. 10937990

[103] Klein DJ, Moore PB, Steitz TA. 2004. The roles of ribosomal proteins in the structure assembly, and evolution of the large ribosomal subunit. J Mol Biol 340:141–177. doi:10.1016/j.jmb.2004.03.076. 15184028

[104] Fox GE, Naik AK. 2004. The evolutionary history of the ribosome, p 92–105. *In* de Pouplana RL (ed), The genetic code and the origin of life. Landes Bioscience, Austin, TX.

[105] Lanyi JK. 1974. Salt-dependent properties of proteins from extremely halophilic bacteria. Bacteriol Rev 38:272–290. doi:10.1128/br.38.3.272-290.1974. 4607500 PMC413857

[106] Rodnina MV, Wintermeyer W. 2016. Protein elongation, co-translational folding and targeting. J Mol Biol 428:2165–2185. doi:10.1016/j.jmb.2016.03.022. 27038507

[107] Thommen M, Holtkamp W, Rodnina MV. 2017. Co-translational protein folding: progress and methods. Curr Opin Struct Biol 42:83–89. doi:10.1016/j.sbi.2016.11.020. 27940242

[108] Cabrita LD, Dobson CM, Christodoulou J. 2010. Protein folding on the ribosome. Curr Opin Struct Biol 20:33–45. doi:10.1016/j.sbi.2010.01.005. 20149635

[109] Tenson T, Ehrenberg M. 2002. Regulatory nascent peptides in the ribosomal tunnel. Cell 108:591–594. doi:10.1016/s0092-8674(02)00669-4. 11893330

[110] Yip MCJ, Shao S. 2021. Detecting and rescuing stalled ribosomes. Trends Biochem Sci 46:731–743. doi:10.1016/j.tibs.2021.03.008. 33966939 PMC8487456

[111] Requião RD, Fernandes L, de Souza HJA, Rossetto S, Domitrovic T, Palhano FL. 2017. Protein charge distribution in proteomes and its impact on translation. PLoS Comput Biol 13:e1005549. doi:10.1371/journal.pcbi.1005549. 28531225 PMC5460897

[112] Ring G, Eichler J. 2004. Membrane binding of ribosomes occurs at SecYE-based sites in the Archaea *Haloferax volcanii*. J Mol Biol 336:997–1010. doi:10.1016/j.jmb.2004.01.008. 15037064

[113] Tirumalai MR, Kaelber JT, Park DR, Tran Q, Fox GE. 2020. Cryo-electron microscopy visualization of a large insertion in the 5S ribosomal RNA of the extremely halophilic archaeon *Halococcus morrhuae*. FEBS Open Bio 10:1938–1946. doi:10.1002/2211-5463.12962. PMC7530397 32865340

[114] Gabdulkhakov A, Nikonov S, Garber M. 2013. Revisiting the *Haloarcula marismortui* 50S ribosomal subunit model. Acta Crystallogr D Biol Crystallogr 69:997–1004. doi:10.1107/S0907444913004745. 23695244

[115] Penczek P, Ban N, Grassucci RA, Agrawal RK, Frank J. 1999. *Haloarcula marismortui* 50S subunit-complementarity of electron microscopy and X-Ray crystallographic information. J Struct Biol 128:44–50. doi:10.1006/jsbi.1999.4157. 10600557

[116] Joiret M, Rapino F, Close P, Geris L. 2020. Ribosome exit tunnel electrostatics. bioRxiv. doi:10.1101/2020.10.20.346684:2020.10.20.346684. 35193250

[117] Nissley DA, Vu QV, Trovato F, Ahmed N, Jiang Y, Li MS, O'Brien EP. 2020. Electrostatic interactions govern extreme nascent protein ejection times from ribosomes and can delay ribosome recycling. J Am Chem Soc 142:6103–6110. doi:10.1021/jacs.9b12264. 32138505 PMC7312765

[118] Lu J, Deutsch C. 2008. Electrostatics in the ribosomal tunnel modulate chain elongation rates. J Mol Biol 384:73–86. doi:10.1016/j.jmb.2008.08.089. 18822297 PMC2655213

[119] O'Leary NA, Wright MW, Brister JR, Ciufo S, Haddad D, McVeigh R, Rajput B, Robbertse B, Smith-White B, Ako-Adjei D, Astashyn A, Badretdin A, Bao Y, Blinkova O, Brover V, Chetvernin V, Choi J, Cox E, Ermolaeva O, Farrell CM, Goldfarb T, Gupta T, Haft D, Hatcher E, Hlavina W, Joardar VS, Kodali VK, Li W, Maglott D, Masterson P, McGarvey KM, Murphy MR, O'Neill K, Pujar S, Rangwala SH, Rausch D, Riddick LD, Schoch C, Shkeda A, Storz SS, Sun H, Thibaud-Nissen F, Tolstoy I, Tully RE, Vatsan AR, Wallin C, Webb D, Wu W, Landrum MJ, Kimchi A, et al. 2016. Reference sequence (RefSeq) database at NCBI: current status, taxonomic expansion, and functional annotation. Nucleic Acids Res 44:D733–D745. doi:10.1093/nar/gkv1189. 26553804 PMC4702849

[120] Schoch CL, Ciufo S, Domrachev M, Hotton CL, Kannan S, Khovanskaya R, Leipe D, Mcveigh R, O’Neill K, Robbertse B, Sharma S, Soussov V, Sullivan JP, Sun L, Turner S, Karsch-Mizrachi I. 2020. NCBI Taxonomy: a comprehensive update on curation, resources and tools. Database (Oxford) 2020:baaa062. doi:10.1093/database/baaa062. 32761142 PMC7408187

[121] Kozlowski LP. 2016. IPC: Isoelectric Point Calculator. Biol Direct 11:55. doi:10.1186/s13062-016-0159-9. 27769290 PMC5075173

