# Supplementary material for: Net Charges of the Ribosomal Proteins of the S10 and spc Clusters of Halophiles Are Inversely Related to the Degree of Halotolerance
Source: Microbiol Spectr. 2021 Dec 15;9(3):e01782-21. doi: 10.1128/spectrum.01782-21 (PMC8672879; doi:10.1128/spectrum.01782-21)
Supplement: SUPPLEMENTAL FILE 1 — Supplemental material. Download SPECTRUM01782-21_Supp_1_seq8.pdf, PDF file, 0.2 MB [file spectrum01782-21_supp_1_seq8.pdf]

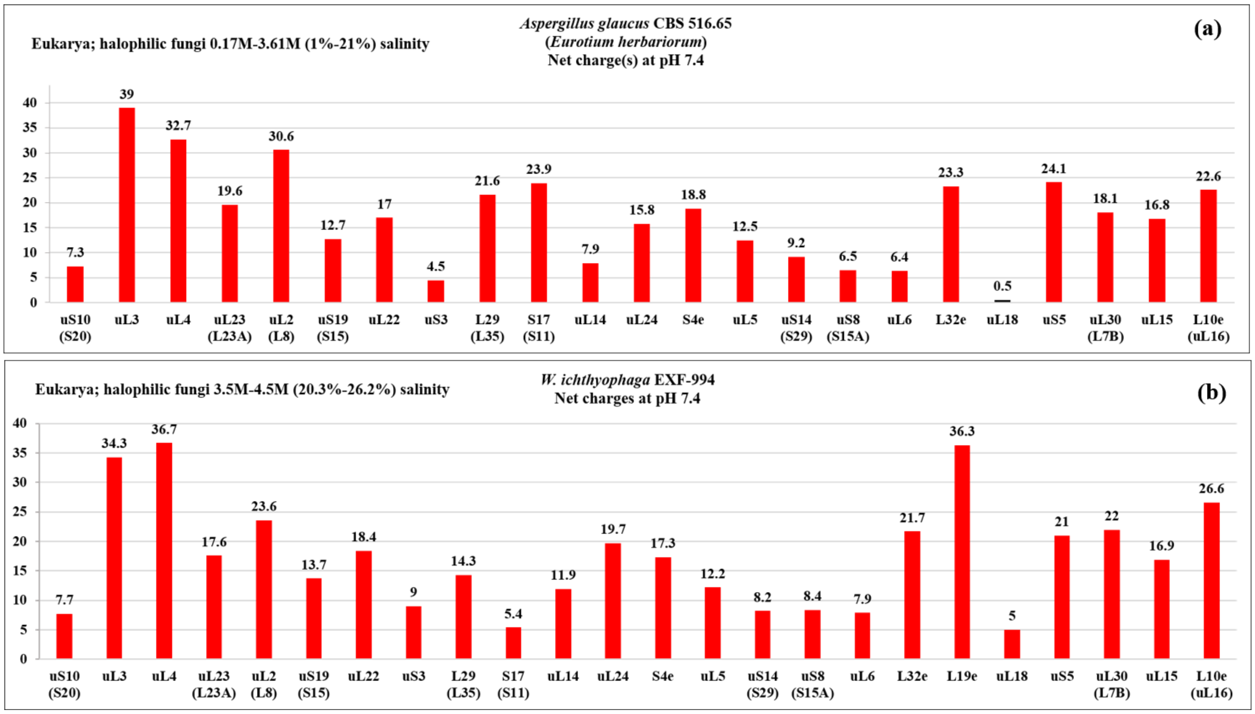

Supplementary Figure 1. Net charges of the ribosomal protein homologs of the *S10-spc* cluster from representative strains of halophilic fungi. The charge value of each protein is shown for each bar; charges greater or lesser than three are in red and black respectively (a) *Aspergillus glaucus* CBS 516.65 (*Eurotium herbariorum*) (1%-21% salt), and (b) *W. ichthyophaga* EXF-994 (20.3%-26.2% salt). L19e in *A. glaucus* is truncated (pseudogene) and hence not shown in the figure.

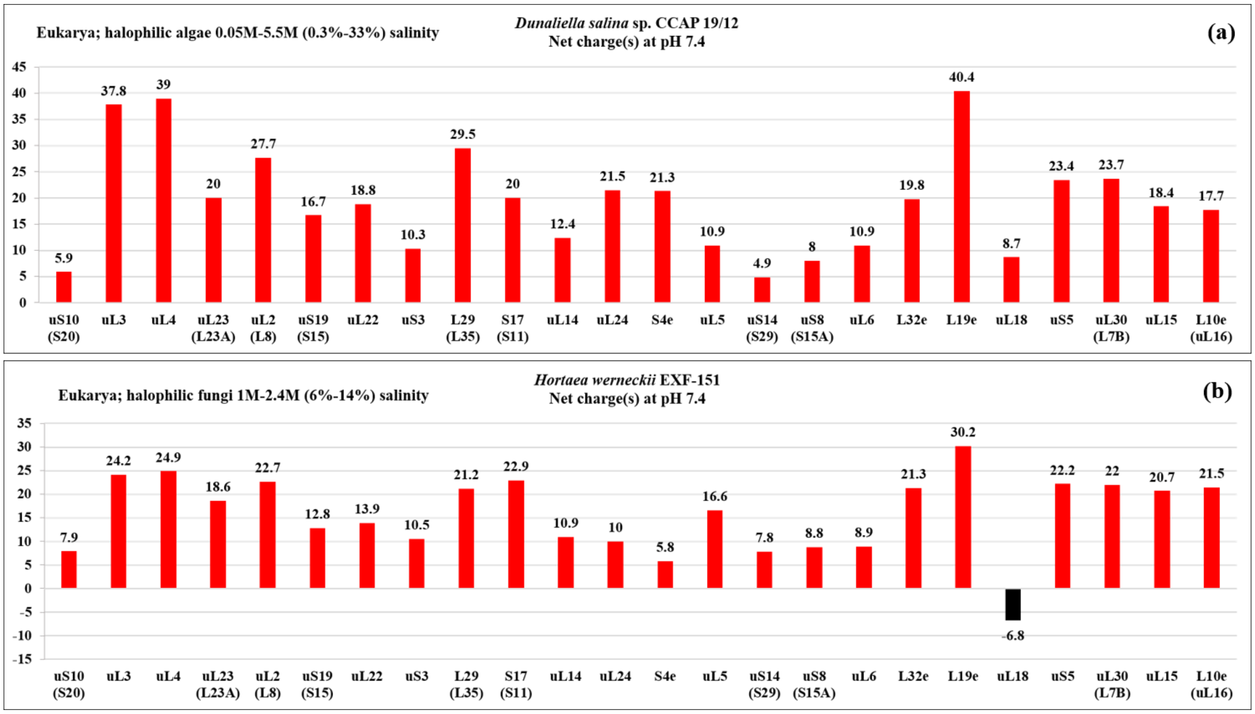

Supplementary Figure 2. Net charges of the ribosomal protein homologs of the S10-spc cluster from representative strains of the halophilic (a) algae - *Dunaliella salina* sp. CCAP 19/12 (0.3%-33% salt), and (b) fungi - *Hortaea werneckii* EXF-151 (6%-14% salt). The charge value of each protein is shown for each bar; charges greater or lesser than three are in red and black respectively.
